# Supplement Use and Increased Risks of Cancer: Unveiling the Other Side of the Coin

**DOI:** 10.3390/cancers16050880

**Published:** 2024-02-22

**Authors:** Parnian Jabbari, Omid Yazdanpanah, David J. Benjamin, Arash Rezazadeh Kalebasty

**Affiliations:** 1 Department of Cell, Molecular and Systems Biology, University of California, Riverside, CA 92521, USA; parnian.jabbari@email.ucr.edu; 2 Division of Hematology and Oncology, Department of Medicine, University of California Irvine Medical Center, Orange, CA 92868, USA; oyazdanp@hs.uci.edu; 3 Hoag Family Cancer Institute, Newport Beach, CA 92663, USA; david.benjamin@hoag.org

**Keywords:** vitamin, supplement, cancer risk, prostate cancer

## Abstract

**Simple Summary:**

This study investigates the overlooked harmful effects of dietary supplements, particularly their potential association with increased cancer risk. In response to a rising trend in supplement consumption, we address the “more is better” mentality prevalent in marketing campaigns by focusing on the long-term and regular use of these supplements. Our findings reveal that beyond known toxicities at high levels, dietary supplements may contribute to increased risk of cancer. We consolidate evidence from studies highlighting the correlation between substantial micronutrient intake, supplement use, and the potential implications for cancer development and mortality. Recognizing the link between supplement consumption and increased cancer risk is crucial for informing consumers and empowering healthcare professionals in how to guide patients more effectively.

**Abstract:**

There is a rising trend in the consumption of dietary supplements, especially among adults, with the purpose of improving health. While marketing campaigns tout the potential health benefits of using dietary supplements, it is critical to evaluate the potential harmful effects associated with these supplements as well. The majority of the scarce research on the potential harmful effects of vitamins focuses on the acute or chronic toxicities associated with the use of dietary supplements. Quality research is still required to further investigate the risks of long-term use of dietary supplements, especially the risk of developing cancers. The present review concentrates on studies that have investigated the association between the risk of developing cancers and associated mortality with the risk of dietary supplements. Such an association has been reported for several vitamins, minerals, and other dietary supplements. Even though several of these studies come with their own shortcomings and critics, they must draw attention to further investigate long-term adverse effects of dietary supplements and advise consumers and healthcare providers to ponder the extensive use of dietary supplements.

## 1. Introduction

With the increasing trends in longevity, the conversation in healthcare is changing from lifespan to that of healthspan [1]. Several genetics and epigenetics studies have been dedicated to finding intrinsic and extrinsic factors that can improve both lifespan and healthspan [2]. Much emphasis has been placed on the importance of a healthy lifestyle and its epigenetic effects in decreasing the risk of several diseases, especially age-related diseases, including cancers [3,4]. A healthy diet and moderate physical activity have been introduced as cornerstones of leading a long, healthy life [5,6]. Among these, micronutrients have received special attention for their role in preventing diseases such as cancers [7]. Following recommendations of a balanced diet and physical activity might be hard, and therefore several shortcuts have been introduced to improve healthspan. A solution to ensure balanced nutrition, especially in the light of concerns for nutrient-depleted food due to soil exploitation [8], is the use of dietary supplements. While dietary supplements have been available for decades, the recent increase in their use is alarming and requires investigations for potential risks associated with their frequent or excessive use. Most of the studies regarding dietary supplements have focused on their benefits, specifically in the light of their effect on a certain health concern. These beneficial effects are one of the driving forces behind the increased consumption of dietary supplements and the multibillion dollar growth in this expanding market. With new reports on the health benefits of various compounds and micronutrients, the nutraceutical industry now offers more than 90,000 dietary supplements to the market, a remarkable increase compared to the 4000 supplements marketed in 1994 [9]. Several marketing campaigns are now providing consumers with information on the health benefits of their dietary supplements. Many of these dietary supplements provide a range of micronutrients to ease the consumption of required micronutrients in one go. However, influential marketing as one of the main contributors to the increased consumption of dietary supplements may lead to the concurrent consumption of dietary supplements [10]. The target consumers may fall into one of two major categories: one group may try to compensate for the lack of a healthy diet by consuming dietary supplements. Even though these individuals may receive daily doses of several micronutrients, they may miss out on the phytochemicals a balanced diet composed of whole foods can offer, which have several health benefits including anticancer properties [11]. Another group of consumers may be those who are obsessed with health. This group might consume more than one multinutrient supplement, leading to the potential consumption of certain micronutrients above the recommended daily doses. The consumption of these micronutrients in the long term has generally been assumed to be safe; however, a limited body of literature suggests an increased risk of various diseases, including cancers, associated with the long-term consumption of dietary supplements. In this review, we provide a critical assessment (Table 1) and a concise overview of the studies that have reported an increased risk of cancers or their associated mortalities with different dietary supplements (Table 2). We aim to encourage more studies focusing on the potential adverse effects of dietary supplements, specifically in light of recent advances in the nutraceutical industry.

## 2. Materials and Methods

This review aimed to investigate the risk of cancer development and/or mortality associated with dietary supplements. A comprehensive search strategy was devised, encompassing relevant studies from PubMed between 1990 and 2023. The search terms included vitamin A, vitamin B1 (or thiamine), vitamin B2 (or riboflavin), vitamin B3 (or niacin or nicotinamide or nicotinic acid or niacinamide), vitamin B5 (or pantothenic acid), vitamin B6 (or pyridoxine), vitamin B7 (or biotin), vitamin B9 (or folic acid/folate), vitamin B12 (or cyanocobalamin), vitamin C (or ascorbic acid), vitamin D (or calciferol), vitamin E (or alpha-tocopherol), vitamin K (or phylloquinone or phytonadione or menaquinone or menaphthone), copper, zinc, selenium, iron, calcium, manganese, and magnesium, combined with terms related to cancer (cancer, cancer risk, or cancer mortality). Herbal supplements were excluded from consideration due to their diverse nature and non-commercially accessible forms. Studies that reported a high risk of cancer or increased cancer mortality associated with a higher consumption of micronutrients or their dietary supplementation as well as high levels of plasma concentration of the micronutrients were included.

## 3. Vitamins

### 3.1. Vitamin A

Vitamin A (FDA daily allowance (DA): 900 mcg) has antioxidant properties and its supplementation is used to boost immune function and prevent respiratory infections [21,22]. Despite in vitro evidence of the protective role of vitamin A against cancer in many instances [23], population-based studies have found an increased risk of certain cancers associated with vitamin A supplementation.

A randomized, double-blind, placebo-controlled clinical trial on 29,133 white male smokers receiving alpha-tocopherol, beta-carotene, alpha-tocopherol + beta-carotene, or placebo was conducted to find associations between circulating levels of vitamin A and prostate cancer development in males. Even though this study did not report a significant correlation between vitamin A intake and serum retinol concentrations, higher serum retinol concentrations were reported to be associated with a higher risk of developing prostate cancer and the aggressiveness of prostate cancer in a dose-dependent manner (hazard ration of 1.19 (95% confidence interval (CI): 1.03, 1.36) for serum retinol concentrations above 685 μg/L compared with that of below 483 μg/L) [12]. Another randomized, double-blind, placebo-controlled chemoprevention trial (Beta-Carotene and Retinol Efficacy Trial, CARET) on 18,314 individuals with a high risk of lung cancer due to a history of smoking or asbestos exposure found a higher incidence of lung cancer (RR of 1.36 (95% CI = 1.07–1.73), cardiovascular disease, and lung cancer mortality (RR of 1.59 (95% CI = 1.13–2.23)) in individuals who received daily doses of 30 mg beta-carotene and 25,000 IU retinyl palmitate (~10 times daily allowance) [13]. Even though the study had to be terminated earlier than scheduled due to the obvious harms of vitamin A supplementation, patients were followed up for five additional years, the results of which showed that adverse effects of the supplementation remained in high-risk individuals, even though the differences between the two groups were no longer significant [24].

### 3.2. Group B Vitamins

Group B vitamins are cofactors for several enzymes involved in metabolism and nucleic acid synthesis [25]. Supplements of group B vitamins are mostly used for improving energy and for addressing their deficiencies in at-risk individuals. High levels of thiamine in the Western diet have been hypothesized to be one of the contributing factors to a higher incidence of cancers in Western countries compared to African and Asian countries, where high levels of thiaminase in the diet degrades thiamine [26]. The effects of thiamine degradation with thiaminase on the proliferation of cancer cells have been studied in vitro and shown to decrease tumor growth through degrading thiamine [27]. Animal studies have shown increased tumor growth with moderate thiamine supplementation [28]. Similar studies have also implicated the interaction of thiamine levels and other dietary aspects, such as high-fat intake, in tumor development [29].

Niacin is required for the synthesis of nicotinamide adenine dinucleotide (NAD+), which is used by several redox enzymes such as poly (ADP-ribose) polymerases (PARPs) and sirtuins for DNA repair [30]. Some studies have shown that certain cancers, especially those with BRCAness, can take advantage of niacin by amplifying nicotinic acid phosphoribosyltransferase (NAPRT) and increasing niacin conversion to NAD+ [31], allowing cancer cells to repair their DNA and ensure the progression of tumorigenesis. Therefore, lowering niacin levels, either through the restriction of dietary intake, or the alteration of the gut microbiota with antibiotics, may reduce the resistance of cancers to treatments targeting the DNA repair of cancer cells [31].

The Vitamins and Lifestyle (VITAL) cohort of 77,118 participants suggested an increased risk of lung cancer in males with a higher intake of pyridoxine and cobalamin. Pyridoxine (DA: 1.7 mg) and cobalamin (DA: 2.4 mcg) supplementation from individual sources were associated with 30% and 40% increased risks of lung cancer in men, respectively (HR, 1.84; 95% CI, 1.01 to 3.36) [15]. Another study in Chile investigating the effects of mandatory flour fortification with folate (DA: 400 mcg) on the incidence of cancers was performed by comparing the rate of cancer hospital admissions in two periods before and after the fortification program. It was found that the risk of colon cancer had increased for specific age groups after folate fortification, with the highest RR of 2.9 for individuals 65–79 years of age (99% CI: 3.25–2.86) [16].

### 3.3. Vitamin E

Vitamin E (DA: 15 mg), mostly known as tocopherol, is a fat-soluble, antioxidant vitamin protecting cells from oxidative stress. Even though several studies have suggested anti-cancer properties for this vitamin due to its scavenging of free radicals [32], some studies have implicated potential harmful effects of vitamin E supplementation with increased risks of cancer development. In the Selenium and Vitamin E Cancer Prevention Trial (SELECT) investigating the effects of selenium and vitamin E supplements on the risk of developing various diseases in healthy men, it was found that vitamin E supplementation alone could increase the risk of prostate cancer in healthy men. A total of 35,533 men were divided into four groups to receive vitamin E supplementation, selenium supplementation, selenium + vitamin E supplementation, or placebo for approximately four years. After a median follow-up of 5.46 years, it was initially reported that vitamin E supplements could slightly increase the risk of prostate cancer in men [33]. A secondary report on the study participants four years after the initial report was indicative of vitamin E supplementation leading to a statistically significant increased risk of developing prostate cancer, with a hazard ratio of 1.17 (99% CI, 1.004–1.36, *p* = 0.008) [14].

## 4. Mineral Supplements

Several inorganic molecules are necessary for the normal function of the body through their involvement in protein structure, maintaining the structure of bones, signal transduction, and maintaining homeostasis, to name a few. High levels of these minerals through supplementary intake can be associated with increased risks of cancer.

### 4.1. Calcium

Calcium (DA: 1300 mg) is most commonly known for its role in bone mineralization, and its physiologic roles expand to signal transduction, the activation of enzymes, and the contraction of muscles [34]. In a prospective cohort study of 30,899 adults of 20 years or older, it was found that the consumption of calcium supplements above tolerable upper intake levels was associated with increased risks of cancer mortality [17]. In this study, individuals provided information on their dietary and supplementary intakes through a questionnaire, and were followed up for a median of 6.1 years. An intake of calcium higher than tolerable levels (2500 mg/day) was associated with higher risk of death from cancer, with a multivariable-adjusted rate ratio of 1.62 (95% CI, 1.07 to 2.45) compared to those with a supplementary intake at or below tolerable levels. Such an observation was not reported for a higher intake of calcium from dietary sources, suggesting a potential effect from the source of nutrients [17].

### 4.2. Selenium

Selenium (DA: 55 mcg) is a trace element required for the activity of certain enzymes in the body, including glutathione peroxidase, which is necessary for the redox balance in the body, as well as deiodinase, necessary for the synthesis of the thyroid hormone [35]. Population-based and genetic studies have looked into the potential effects of selenium on the risk of cancer, or risk of mortality from cancer. Some studies have looked into the association of selenium supplement use with cancer recurrence and mortality, along with overall mortality [35]. In one study, 4459 men with non-metastatic prostate cancer were followed up for a median of 8.9 years. The use of selenium at 140 mcg/day, which is almost triple the amount of suggested daily use, was associated with higher mortality rates (hazard ratio (HR): 2.32, 95% CI: 1.47 to 3.65) due to prostate cancer in a dose-dependent manner [18].

### 4.3. Zinc

Zinc (DA:11 mg) is a trace element that is virtually limited to the intracellular compartment in multicellular organisms and plays structural and functional roles in proteins, including transcription factors; hence, it is an important regulator of cell division, and potentially cancer progression [36]. In a cohort of 47,240 men from the Health Professionals Follow-up Study, participants’ zinc supplement use was inquired into at the time of enrollment, and biennially for the duration of the follow up. Participants were followed up for a median of 28.3 years between 1986 and 2016. It was found that participants with zinc supplement consumption higher than 75 mg/day had a higher risk of developing lethal (HR:1.76, 95% CI: 1.16–2.66) or aggressive (HR: 1.80, 95% CI: 1.14, 2.84) prostate cancer. Similarly, participants who had more than 15 years of zinc supplement consumption had a higher chance of developing lethal (HR: 1.91, 95% CI: 1.28–2.85) or aggressive (HR: 1.55, 95% CI; 1.03–2.33) prostate cancer [19].

## 5. Probiotics

Several studies have investigated the role of gut microbiota in health and various diseases. The presence of these bacteria is important for the host’s physiology. As an instance, a sterile gut such as that of infants before the introduction of maternal and environmental bacteria is associated with hemorrhage due to a lack of vitamin K synthesis by the gut microbiota [37]. An altered gut microbiota has been associated with resistance to chemo drugs [38]. Furthermore, dysbiosis of the gut bacteria has been found to be associated with several chronic inflammatory and autoimmune disorders, implicating the role of these bacteria in the regulation of the immune system [37,39].

The symbiotic bacteria in the gut rely on dietary fiber to survive and produce nutrients for the host, such as short-chain fatty acids [37]. The Western diet, high in amino acids and fats, promotes the growth of bacteria that can survive on these macromolecules and wipe out the beneficial effects of a high-fiber diet on the gut microbiota [40]. As a solution to this problem, “probiotics”, composed mainly of bacteria in the Lactobacillus and Bifidobacterium genera, have been made commercially available to reintroduce these bacteria. While most studies have focused on the benefits of probiotic supplementation [41], recent findings on the interaction of Lactobacilli and macrophages bring new insights into the possible harmful effects of probiotic supplements. In the recent study by Hezaveh et al., it was found that reducing the ratio of Lactobacilli in the gut microbiome with ampicillin could reduce the tumor weight in mice [42]. This observation has been attributed to the ability of Lactobacilli to activate aryl hydrocarbon receptors (AhR) of macrophages, which is associated with poor prognosis and a higher tumor grade [43]. Given the role of the gut microbiota in regulating immune response and tumor burden [44], it is important to be vigilant when altering the gut microbiome through diet or supplementation.

## 6. Miscellaneous

### 6.1. Omega-3 Fatty Acids/Fish Oil Supplements

Omega-3 fatty acids are unsaturated fatty acids found in several plant sources, such as leafy greens, nuts, beans, and seeds, as well as in fish and fish products [45]. Another source of omega-3 fatty acids is dietary supplements, some of which are available over the counter, while others, such as icosapent ethyl and omega-3-acid ethyl esters, are available only through prescription. While these supplements have been suggested or prescribed along with dietary modifications to improve the lipid profile, recent evidence suggests potential side effects beyond a fishy smell. A subpopulation of the SELECT trial, consisting of 834 men who developed prostate cancer during the first 6 years of the trial, was compared to a matched sub-cohort of 1393 men in terms of omega-3 fatty acids and risk of prostate cancer. Participants’ fatty acid levels were divided into quartiles, and a comparison of the highest quartile of long-chain omega-3 polyunsaturated fatty acids with its lowest quartile showed an increased risk of low-grade (44%), high-grade (71%), and total prostate cancer (43%) [20]. These findings are supported by several other studies investigating the association between omega-3 fatty acids and the risk of prostate cancer, as summarized by Brasky et al. [46,47,48].

### 6.2. Nicotinamide Supplements

As mentioned earlier in the review, NAD+ is required by several redox enzymes as well as for the protection and repair of DNA. Studies have focused on the role of this molecule and other nicotinamide derivatives and sirtuin-activating compounds in various disorders, specifically in degenerative disorders [49]. As the organisms age, their cellular reservoir of NAD+ is diminished through the increased function of molecules such as CD38 [49]. Studies have found benefits of nicotinamide derivatives and sirtuin-activating compounds for longevity and in combatting age-related disorders by replenishing the cellular NAD+ reservoir [50]. However, assuming aging and cancer to be two ends of the same spectrum [51], it is not surprising that these compounds have potentially pro-cancer effects. Even though research is still in its infancy regarding the role of these compounds and most studies on them are in vitro or in vivo animal studies, some studies have found evidence that these molecules can promote cancer [52].

Cancer cells have a high energy demand and a large reservoir of NAD+ to meet their energy requirements [53]. It is plausible that cells reduce their NAD+ reservoirs as an anti-cancer strategy. The accumulation of genetic mutations that is a normal process of aging predisposes the organism to develop cancer [54]. Cancerous cells have a lower chance of proliferation when there is a small NAD+ pool compared to a replenished NAD+ reservoir status through supplementation due to restricted energy production. Additionally, in vitro studies have found that cancer cells release NAD+ into the culture medium, suggesting an autocrine or paracrine activity for this molecule [55]. In an in vivo animal study, the effects of Nicotinamide riboside (NR), a common supplement to increase NAD+ levels, were found to have a high uptake by triple-negative cancer cells in vitro, and mice inoculated with cancer cells showed higher rates of tumor formation when on an NR-rich diet [56]. Even though this evidence may not be enough to conclude the potential harmful effects of NAD+ supplements, it is enough to implement caution while more studies investigate the in vivo effects of these compounds, especially in long-term use.

## 7. Discussion

There is a remarkable body of literature regarding the effects of micronutrients on health and adverse effects of their deficiencies from in vitro, in vivo, population-based, or clinical studies. This manuscript focused on micronutrients for which supplementation was associated with increased risk of cancers or their associated mortalities, as summarized in Table 3.

## 8. Conclusions

In an effort to eliminate potential deficiencies of these micronutrients, several of them are available through dietary supplements. While the supplementation of certain micronutrients is necessary for at-risk groups, there is not a general recommendation or guideline for the supplementation of these micronutrients for healthy individuals. However, the consumption of dietary supplements is on the rise to prevent potential deficiencies or to boost health. Micronutrient supplementation is considered to be a safe and beneficial practice as there are not many studies reporting adverse effects other than potential toxicities. Several considerations must be taken when interpreting the results of these studies. Most of the studies on the health benefits of the supplementation of micronutrients, especially in vitro studies, used highly concentrated or purified preparations of the micronutrients delivered directly or through a safe vehicle. Dietary supplements include several additives for different purposes, which can be associated with adverse effects on their own [57], specifically in the light of a lack of regulation by the Food and Drug Administration (FDA). Other considerations regarding these studies are the dosage and study population. Even though ensuring adequate or supplemented levels of certain vitamins can deliver health benefits for at-risk groups, they can have adverse effects for other, potentially overlapping groups. Several of the studies summarized herein report such adverse effects with concurrent smoking or exposure to certain environmental/professional exposures. Another important determinant of health benefits or adverse effects of micronutrient supplementation is the time of exposure to high doses of them. A long-term follow up on consumers of dietary supplements, as reported here, may reveal adverse effects that can outweigh their potential benefits. With the increasing trends in the use of dietary supplementations, it is important that more population-based studies and clinical trials investigate the potential adverse effects of their long-term use, including cancers. This is especially required for dietary supplements promising rejuvenation and fighting senescence, as they may tip the balance toward oncogenesis.

## Figures and Tables

**Table 1:** Standardized quality assessment of reviewed studies.

Clinical Trials							
Study	Randomized and Blinded?	Allocation Concealment	Baseline Group Similarities	Dropout Rate	Intervention Control	Adherence Assurance	Sample Size and Power Reported
Mondul et al. [12]	Yes	Yes	Yes	NR	Yes	Yes	Yes
Omenn et al. [13]	Yes	Yes	Yes	NR	Yes	Yes	Yes
Klein et al. [14]	Yes	Yes	Yes	NR	Yes	Yes	Yes
**Cohorts/Cross-Sectional Studies**							
**Study**	**Clear Objective** **and Study** **Population**	**Sample Size** **and Power** **Reported**	**Sufficient** **Follow-Up** **Period**	**Leveling** **of Exposure**	**Exposure** **Measured** **before** **Outcome**	**Multiple** **Exposure** **Assessments**	**Confounding** **Variables** **Considered**
Brasky et al. [15]	Yes	Yes	Yes	Yes	Yes	Yes	Yes
Hirsch et al. [16]	Yes	NR	NA	CD	NA	No	Yes
Chen et al. [17]	Yes	Yes	Yes	Yes	Yes	Yes	Yes
Kefield et al. [18]	Yes	Yes	Yes	Yes	Yes	Yes	Yes
Zhang et al. [19]	Yes	Yes	Yes	Yes	Yes	Yes	NR

CD: Cannot Determine, NA: Not Applicable, NR: Not Reported.

**Table 2:** Risk of cancer or cancer mortality associated with dietary supplements.

Nutrient	Study Design	Study Population	Follow Up	Supplement Intake	Risk of Cancer (*p* < 0.05)	Ref.
Vitamin A	Double Blind RCT	29,133 White males between 1985 and 1988	5–8 years	(1) α-tocopherol 50 mg/day), (2) β-carotene (20 mg/day), (3) both supplements, (4) placebo	Higher serum retinol at baseline led to higher risk of PCA incidence. HR = 1.19 (95% CI: 1.03–1.36)	[12]
					Sustained high exposure to serum retinol led to greatest risk of PCA incidence. HR = 1.31 (95% CI: 1.08–1.59)	
Vitamin A	Double Blind RCT	18,314 Men and women, high risk for lung cancer	24 months	(1) combination of 30 mg beta-carotene and 25,000 IU vitamin A daily, (2) placebo	Beta-carotene and Vit A supp. resulted in excess lung CA incidence. HR = 1.36 (95% CI: 1.07–1.73)	[13]
					Beta-carotene and vit A resulted in excess lung CA mortality. HR = 1.59 (95% CI: 1.13–2.23)	
Vitamin B6	Cohort Study	77,118 Men and women, 50 to 76 years between 2000 and 2002	6 years	10 years average daily dose (mg/d) (1) non-user, (2) 0.4–1.4, (3) 1.4–3.0, (4) 3.0–20, (5) >20	Higher risk of lung CA after 10 y Vit B6 supp. HR = 1.82 (95% CI: 1.25–2.65)	[15]
Vitamin B9	Population-Based Study	Cancer discharge trends in Chile, 1992–1996 vs. 2001–2004 (before and after flour folic acid fortification)	N/A	Average consumption of 185 g of flour containing 410 μg folic acid	B9 supp. program resulted in additional risk of colon cancer HR = 2.9 (99% CI: 2.86–3.25)	[16]
Vitamin B12	Cohort Study	77,118 participants 50–76 years, between 2000 and 2002	6 years	10 years average daily dose (µg/d) (1) non-user, (2) 0.1–5.00, (3) 5.01–25.00, (4) 25.01–55.00, (5) >55.00	Higher risk of lung CA after 10 y Vit B12 supp. HR = 1.98 (95% CI: 1.32–2.97)	[15]
Vitamin E	Double Blind RCT	35,533 men between 2001 and 2004	7–12 years	(1) Oral selenium (200 μg/d, (2) Vitamin E (400 IU/d), (3) both agents, (4) placebos	Increased risk of PCA Development with Vit E supp. HR = 1.17 (99% CI: 1.004–1.36)	[14]
Selenium	Prospective Cohort Study	4459 men, 40–75 years initially diagnosed with non-metastatic prostate cancer	7.8 years	Selenium supplement μg/day: (1) non-user (2) 1–24 (3) 25–139 (4) >140	Increased PCA mortality, highest of >140 μg/day selenium supp. HR = 2.60 (95% CI: 1.44–4.70)	[18]
Zinc	Prospective Cohort Study	47,240 men, 40–75 years between 1986 and 2016	28.3 years	Zinc (mg/d): (1) non-user, (2) 1–24, (3) 25–74, (4) ≥75	>75 mg/day zinc supp led to increased risk of aggressive PCA. HR = 1.80 (95% CI: 1.19–2.73)	[19]
					>15 years zinc supp led to increased risk of PCA mortality. HR = 1.91 (95% CI: 1.28–2.85)	
Omega-3	Case Cohort Design Nested within SELECT trial	834 men with PCA and 1393 men selected randomly at baseline	4.5 years	(1) Oral selenium (200 μg/d, (2) Vitamin E (400 IU/d), (3) both agents, (4) placebos	Higher PCA risk in men with high Omega-3 serum level. HR = 1.43 (95% CI: 1.09–1.88)	[20]

Randomized controlled clinical trial (RCT), PCA: Prostate Cancer, Vit: Vitamin, CA: Cancer, supp: supplementation, N/A, not applicable, SELECT trial (Selenium and Vitamin E Cancer Prevention Trial).

**Table 3:** Summary of cancers associated with micronutrient supplementation.

Supplement	Associated Higher Risk	References
Vit A	Prostate cancer and lung cancer	[12,13]
Vit B6	Lung cancer	[15]
Vit B9	Colon cancer	[16]
Vit B12	Lung cancer	[15]
Vit E	Prostate cancer	[14]
Selenium	Prostate cancer	[18]
Zinc	Prostate cancer	[19]
Omega-3	Prostate cancer	[20]
